# Systematic Characterization of the *OSCA* Family Members in Soybean and Validation of Their Functions in Osmotic Stress

**DOI:** 10.3390/ijms231810570

**Published:** 2022-09-12

**Authors:** Congge Liu, Hong Wang, Yu Zhang, Haijing Cheng, Zhangli Hu, Zhen-Ming Pei, Qing Li

**Affiliations:** 1 Longping Branch, College of Biology, Hunan University, Changsha 410125, China; 2 College of Life Sciences and Oceanography, Shenzhen University, Shenzhen 518060, China; 3 State Key Laboratory of Rice Biology, China National Rice Research Institute, Hangzhou 311401, China; 4 Department of Biology, Duke University, Durham, NC 27708, USA

**Keywords:** osmotic stress, osmosensor, calcium, *OSCA*, soybean

## Abstract

Since we discovered OSCA1, a hyperosmolarity-gated calcium-permeable channel that acted as an osmosensor in *Arabidopsis*, the *OSCA* family has been identified genome-wide in several crops, but only a few *OSCA* members’ functions have been experimentally demonstrated. Osmotic stress seriously restricts the yield and quality of soybean. Therefore, it is essential to decipher the molecular mechanism of how soybean responds to osmotic stress. Here, we first systematically studied and experimentally demonstrated the role of *OSCA* family members in the osmotic sensing of soybean. Phylogenetic relationships, gene structures, protein domains and structures analysis revealed that 20 *GmOSCA* members were divided into four clades, of which members in the same cluster may have more similar functions. In addition, *GmOSCA* members in clusters III and IV may be functionally redundant and diverged from those in clusters I and II. Based on the spatiotemporal expression patterns, *GmOSCA1.6*, *GmOSCA2.1*, *GmOSCA2.6*, and *GmOSCA4.1* were extremely low expressed or possible pseudogenes. The remaining 16 *GmOSCA* genes were heterologously overexpressed in an *Arabidopsis osca1* mutant, to explore their functions. Subcellular localization showed that most GmOSCA members could localize to the plasma membrane (PM). Among 16 *GmOSCA* genes, only overexpressing *GmOSCA1.1*, *GmOSCA1.2*, *GmOSCA1.3*, *GmOSCA1.4*, and *GmOSCA1.5* in cluster I could fully complement the reduced hyperosmolality-induced [Ca^2+^]_i_ increase (OICI) in *osca1*. The expression profiles of *GmOSCA* genes against osmotic stress demonstrated that most *GmOSCA* genes, especially *GmOSCA1.1*, *GmOSCA1.2*, *GmOSCA1.3*, *GmOSCA1.4*, *GmOSCA1.5*, *GmOSCA3.1*, and *GmOSCA3.2*, strongly responded to osmotic stress. Moreover, overexpression of *GmOSCA1.1*, *GmOSCA1.2*, *GmOSCA1.3*, *GmOSCA1.4*, *GmOSCA1.5*, *GmOSCA3.1*, and *GmOSCA3.2* rescued the drought-hypersensitive phenotype of *osca1*. Our findings provide important clues for further studies of *GmOSCA*-mediated calcium signaling in the osmotic sensing of soybean and contribute to improving soybean drought tolerance through genetic engineering and molecular breeding.

## 1. Introduction

As sessile organisms, plants need to adapt to the changing environments around them, especially biotic and abiotic stresses, to sustain their growth and development. Common abiotic stresses include drought, salinity, waterlogging, high temperature, cold, acid rain, and heavy metal pollution. Among these, drought and salinity, which both induce osmotic stress in plant cells, are the two most prevalent environmental stresses that affect the geographical distribution of plants in nature and restrict crop growth and yield in agriculture [1]. It is estimated that global maize and wheat yields are reduced by about 40% and 21% due to drought stress, respectively [2]. In addition, more than one-third of the irrigated lands in the world are affected by salinization due to seawater intrusion and poor-quality agricultural management practices [3], which seriously threatens crop cultivation and food security. To reduce the damages of osmotic stress such as drought and salinity, plants have evolved different resistance mechanisms at the morphological, physiological, cellular, biochemical, and molecular levels [4].

Although the signal transduction pathway of plant response to osmotic stress is very complex, it can be divided into three stages in general, including signal perception, signal transduction, and adaptive responses to stress signals [5,6,7]. Firstly, plants perceive osmotic stress signals through their specific receptors. After the initial perception, the second messengers such as calcium (Ca^2+^), reactive oxygen species (ROS), and inositol phosphates are generated during the early signaling responses, which further modulate the intracellular free Ca^2+^ concentrations ([Ca^2+^]_i_) and trigger [Ca^2+^]_I_ fluctuation. The [Ca^2+^]_i_ fluctuation is sensed by Ca^2+^ sensors, which then interact with their corresponding partners to activate a phosphorylation cascade. The signal cascade leads to altered expression of major stress-responsive genes and induced abscisic acid (ABA), an important plant stress signaling hormone, which accumulates during downstream signaling. Finally, the products of these stress genes result in plant adaptation to unfavorable conditions.

To date, the signal transduction process of osmotic stress in plants has been thoroughly studied, and the transcriptional regulation of osmotic-stress-responsive gene expression is mainly governed by ABA-dependent and ABA-independent pathways [8]. The cis-element of ABA-responsive element (ABRE), and a class of transcription factors, the ABRE-binding proteins/ABRE-binding factors (AREBs/ABFs), play critical roles in ABA-dependent gene expression. Under osmotic stress, AREBs/ABFs are phosphorylated and activated by SNF1-related kinase 2s (SnRK2s) in an ABA-dependent manner, then bind to ABRE in the promoter regions of target genes, and, thereby, induce the stress-responsive genes’ expression [9,10,11]. In contrast, the cis-element of dehydration-responsive element/C-repeat (DRE/CRT) and DRE/CRT-binding protein 2 (DREB2) transcription factors have critical functions in ABA-independent gene expression. During osmotic stress, *DREB2* transcripts are highly induced, and DREB2 proteins are also stabilized, then bind to DRE, and, consequently, activate their target genes’ expression [8,12,13]. In addition, there is a crosstalk between ABA-independent and ABA-dependent pathways [8]. Besides these master regulators, WRKY, MYB, MYC, and NF-Y transcription factors are also involved in osmotic response and tolerance [14].

In contrast to processes of signal transduction and adaptive responses to stress signals, the molecular mechanisms underlying how plants sense osmotic stress are not well understood. In *Arabidopsis*, OSCA1, a hyperosmolarity-gated calcium-permeable channel that acted as an osmosensor, was identified using a calcium-imaging-based forward-genetic screen in our previous study [15]. It is the first and only known class of plant osmosensors. OSCA1 was located in the plasma membrane (PM), and its dysfunctional *osca1* mutant exhibited reduced hyperosmolality-induced [Ca^2+^]_i_, increased (OICI), decreased root growth, and showed defective leaf transpiration under hyperosmotic stress. Phylogenetic analyses revealed that land plants had four ancient clades of *OSCA1* homologs and that *Arabidopsis* contains 15 *OSCAs* [15]. For this reason, *OSCA1* was named *AtOSCA1.1*, and the other members were called *AtOSCA1.2-1.8*, *AtOSCA2.1-2.5*, *AtOSCA3.1*, and *AtOSCA4.1*, according to their relative distance from *AtOSCA1.1*. *AtOSCA1.2* (also called *AtCSC1*), which shares the highest homology with *AtOSCA1.1*, also encodes a hyperosmolarity-gated calcium-permeable channel and responds to hyperosmotic stress [16]. In addition, AtOSCA1.3, located in the PM, is a BIK1-activated calcium-permeable channel and is specifically required for plant stomatal immunity [17]. However, the molecular functions of other *Arabidopsis OSCA* members are currently unknown.

As the first and only kind of osmosensors to be reported in plants, the *OSCA* gene family has been genome-wide identified and analyzed in several crops, such as rice [18], maize [19,20], and wheat [21]. However, only a few *OSCA* family members’ functions have been experimentally demonstrated. In addition, there is no systematic study on the roles of *OSCA* family members in soybean. As an important crop to provide seed protein and edible oil, soybean is vulnerable to osmotic stress, leading to a severe decline in soybean yield and quality. Therefore, studying the molecular mechanism of osmotic stress sensing and response in soybean is of great significance. To explore whether the soybean *GmOSCA* family members are involved in osmotic stress perception or response, we performed a genome-wide identification and comprehensive characterization of *GmOSCA* members through phylogenetic relationships, gene structures, protein domains and structures, and spatiotemporal and stressed expression profiles in this study. More importantly, the functions of *GmOSCA* members were investigated by the heterologous expression of them in an *osca1* mutant. As a result, a total of 20 *GmOSCA* members were identified in the soybean genome, and they were classified into four classes based on phylogenetic analysis. Among them, only *GmOSCA1.1*, *GmOSCA1.2*, *GmOSCA1.3*, *GmOSCA1.4*, and *GmOSCA1.5* could functionally complement the reduced OICI phenotype in *osca1* under hyperosmotic stress. In addition, *GmOSCA1.1*, *GmOSCA1.2*, *GmOSCA1.3*, *GmOSCA1.4*, *GmOSCA1.5*, *GmOSCA3.1*, and *GmOSCA3.2* were significantly induced by osmotic stress, and their ectopic expression in *osca1* conferred enhanced drought tolerance on transgenic plants. Our findings will improve the understanding of the genetic and molecular basis for osmotic stress perception in soybean and provide valuable gene resources for improving soybean drought tolerance through genetic engineering and molecular breeding.

## 2. Results

### 2.1. Phylogenetic Tree, Gene Structures, Protein Domains and Structures of GmOSCA Genes

To identify all of the *OSCA* family members in soybean, the 15 AtOSCA protein sequences were used as baits to search the soybean genome (Wm82.a2.v1) in Phytozome (https://phytozome.jgi.doe.gov/pz/portal.html, accessed on 15 August 2022). Ultimately, 20 *GmOSCA* genes were screened after removing candidates with low protein homology, candidates without typical RSN1_7TM domain of OSCA family proteins, and candidates not localized on chromosomes. The 20 *GmOSCA* members were mapped onto the 15 chromosomes in the soybean genome (Supplementary Materials Table S1). Their proteins ranged from 500 to 803 amino acids in length, varied between 57,229.64 to 92,256.79 Da in relative molecular weight, and the predicted isoelectric points ranged from 6.35 to 9.38 (Supplementary Materials Table S1). These proteins varied widely, with 13.3% to 98.5% pairwise sequence identity (Supplementary Materials Figure S1), suggesting that they may have divergent functions.

**Figure 1:**
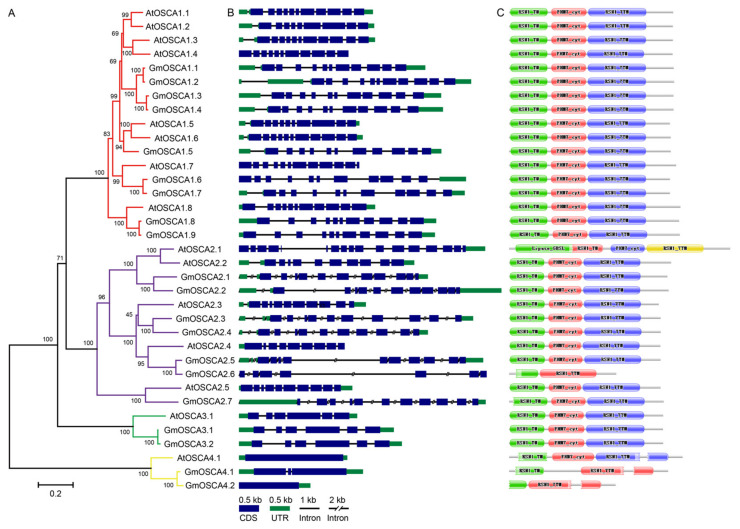
The phylogenetic relationships, gene structures, and conserved domains of *OSCA* genes from *Arabidopsis* and soybean. (**A**) A neighbor-joining tree was constructed using 35 OSCA protein sequences by MEGA 6.0 software with 1000 bootstrap replications. Clusters I, II, III, and IV were marked with red, blue, green, and yellow colors, respectively. (**B**) The exon-intron structures of *OSCA* genes. (**C**) The conserved domains in OSCA proteins.

To investigate the evolutionary relationships of *GmOSCA* members, a neighbor-joining phylogenetic tree using the OSCA protein sequences from *Arabidopsis* and soybean was constructed (Figure 1A). Similar to the classification of AtOSCA members, 20 GmOSCA proteins were divided into four clades, according to the bootstrap values and phylogenetic topology. Based on the similarity with the corresponding AtOSCA proteins, they were named *GmOSCA1.1* to *1.9* in cluster I, *GmOSCA2.1* to *2.7* in cluster II, *GmOSCA3.1* and *GmOSCA3.2* in cluster III, and *GmOSCA4.1* and *GmOSCA4.2* in cluster IV, respectively.

As a paleo-polyploid crop, soybean has undergone at least two whole-genome duplication (WGD) events, thereby generating a highly duplicated genome, with nearly 75% of the genes showing multi-copies [22]. The collinearity analysis revealed that all *GmOSCA* genes, except for *GmOSCA1.5* and *GmOSCA2.7*, had duplicated counterparts (Supplementary Materials Figure S2). The generation of duplicated genes could facilitate gene evolution through nonfunctionalization, neofunctionalization, and subfunctionalization.

The exon/intron structure divergence in duplicate genes played a crucial role during the evolution of some gene families [23]. Therefore, we compared the exon/intron structures of *OSCA* genes from *Arabidopsis* and soybean (Figure 1B). It showed that most *OSCA* genes contained multiple exons (>5), except for cluster IV. Moreover, the number of exons in the same cluster was almost identical. For instance, there were 9~11 exons in clusters I and II, whereas fewer exons were included in cluster III.

As the primary executor of biological functions, proteins with similar motifs and structures in the same gene family are likely to have the same functions. Thus, the conserved domains and three-dimensional structures of OSCA proteins from *Arabidopsis* and soybean were predicted and compared. The results showed that these OSCA proteins not only contained the typical RSN1_7TM domain (calcium-dependent channel, 7TM region) at the carboxyl end but also had the RSN1_TM domain (late exocytosis, associated with Golgi transport) at the amino end and the PHM7_cyt domain (cytosolic domain of 10TM putative phosphate transporter) in the middle, except for GmOSCA2.6, GmOSCA4.1, and GmOSCA4.2 (Figure 1C). As expected, the three-dimensional structures of OSCAs proteins in clusters III and IV were significantly different from those in clusters I and II (Supplementary Materials Figure S3), implying that the OSCA members in clusters III and IV were likely to have functionally diverged from those in clusters I and II.

In sum, the comparative analysis of homologs between soybean and *Arabidopsis* contributed to exploring the *OSCA* functions in soybean. The *OSCA* members in the same cluster might have more similar functions. In addition, *GmOSCA* genes in clusters III and IV might be functionally redundant because they had more copies than those in *Arabidopsis*.

### 2.2. Spatiotemporal Expression Patterns of GmOSCA Genes

**Figure 2:**
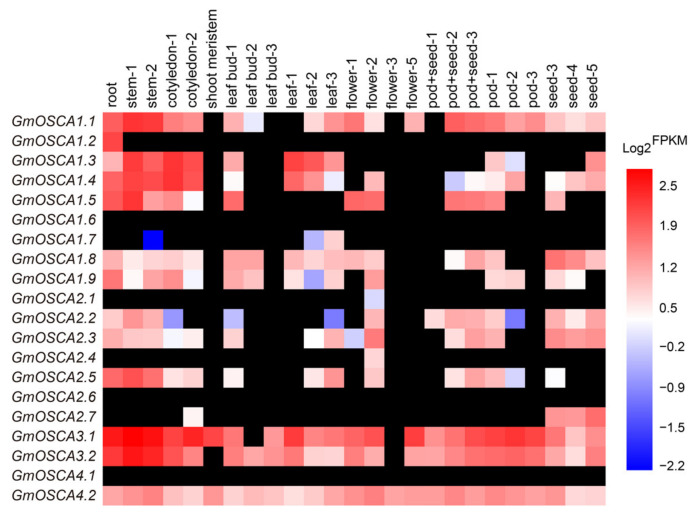
The spatiotemporal expression profiles of *GmOSCA* genes in soybean. The size number behind each sample indicated earlier to later developmental stages with the same sample. Gradient color blocks represented log2-transformed FPKM values. The black blocks indicated that no FPKM value was available.

Gene expression profiles are helpful in predicting gene functions. The previously reported Illumina RNA-seq raw data [24] were used to mine the expression profiles of *GmOSCA* genes in 25 samples (Figure 2). It showed that the expression patterns of *GmOSCA* genes were very different, even those genes from the same cluster, indicating that *GmOSCA* genes might function in different tissues at different developmental periods. For instance, *GmOSCA3.1*, *GmOSCA3.2*, and *GmOSCA4.2* showed high gene expression levels in almost every sample, suggesting they might play important roles throughout soybean growth and development. In contrast, *GmOSCA1.6*, *GmOSCA2.6*, and *GmOSCA4.1* were not detected in all samples, indicating that they were potential pseudogenes. Additionally, *GmOSCA1.2*, *GmOSCA1.7*, *GmOSCA2.1*, *GmOSCA2.4*, and *GmOSCA2.7* exhibited tissue-specific expression patterns, which were only detected in some specific tissues, implicating that they might have served a unique function at a particular time and place. Moreover, *GmOSCA1.1*, *GmOSCA1.3*, *GmOSCA1.4*, *GmOSCA1.5*, *GmOSCA1.8*, *GmOSCA1.9*, *GmOSCA2.2*, *GmOSCA2.3*, and *GmOSCA2.5* were expressed in most tissues with differential expression patterns. Apart from the RNA-seq data by Shen et. al, the expression profiles of *GmOSCA* genes were also explored in nine tissues, using the online soybean gene expression database in Phytozome (https://phytozome.jgi.doe.gov/pz/portal.html, accessed on 15 August 2022) The results showed that the expression patterns of *GmOSCA* genes were similar in the two-transcriptome data, except for *GmOSCA1.2* and *GmOSCA4.1* (Figure 2 and Supplementary Materials Figure S4).

### 2.3. GmOSCA Proteins Are Mainly Located in Membrane Systems

**Figure 3:**
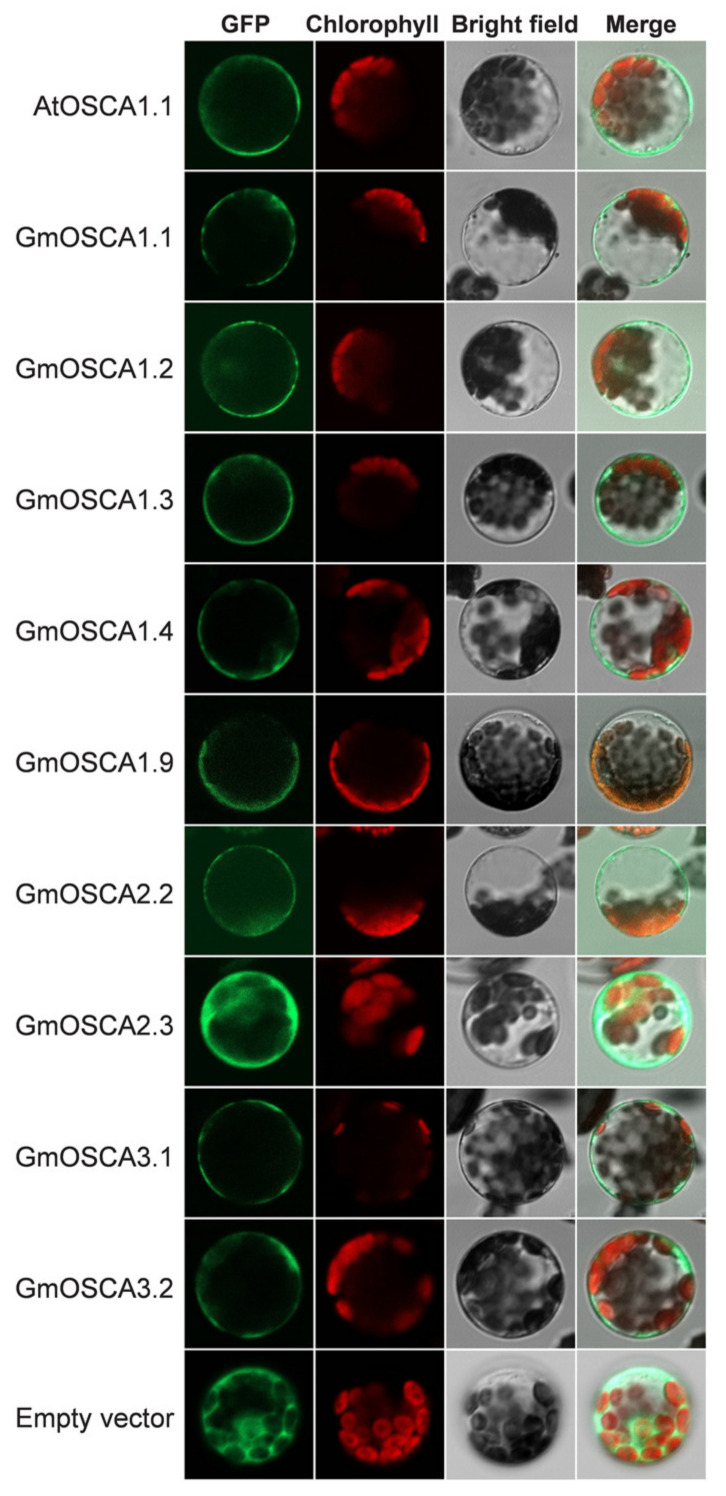
The subcellular localization of GmOSCA proteins.

AtOSCA1.1 and AtOSCA1.2 have multiple transmembrane helices and are localized in the PM to form calcium-permeable channels [15,16]. Therefore, as Ca^2+^ channels, OSCA family members need to meet at least two traits: multiple transmembrane helices and localization on membrane structures. To determine whether GmOSCA proteins have these two characteristics, the transmembrane helices and subcellular localizations were predicted using MemPype (http://mu2py.biocomp.unibo.it/mempype/). It showed that all of the GmOSCA members have at least seven transmembrane helices and could be localized in the membrane system (Supplementary Materials Table S1), implicating their potential as Ca^2+^ channels. To further confirm the prediction accuracy of the subcellular localization, some GmOSCA members were selected to conduct subcellular localization experiments, using the protoplast transient transformation system. The results revealed that GmOSCA1.1 to GmOSCA1.4 and GmOSCA1.9 in cluster I, GmOSCA2.2 and GmOSCA2.3 in cluster II, and GmOSCA3.1 and GmOSCA3.2 in cluster III could localize to the PM, which was similar to the subcellular localization of AtOSCA1.1 (Figure 3). Notably, these OSCA proteins might be localized elsewhere besides PM, especially for GmOSCA2.3 (Figure 3).

### 2.4. Overexpression of Some GmOSCA Members Rescues Decreased OICI in osca1 Mutant

**Figure 4:**
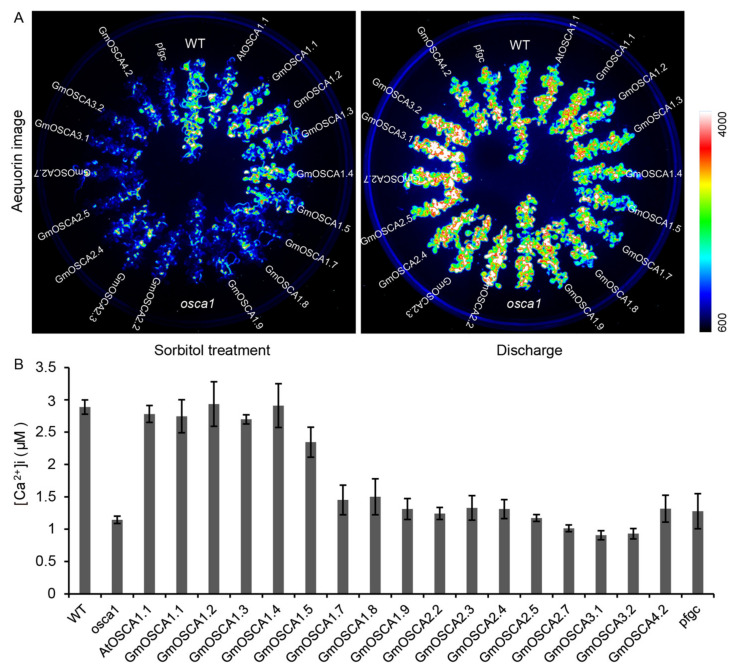
The calcium-imaging phenotype of *GmOSCA*s transgenic *Arabidopsis* seedlings. (**A**) Aequorin bioluminescence-based imaging of seedlings treated with 600 mM sorbitol (left) and discharge solution (right), respectively. (**B**) Quantification of increased [Ca^2+^]_i_ data from representative experiments (mean ± SD, n = 4 pools, 20–22 seedings per pool).

To further explore the biological functions of *GmOSCA* genes, except for *GmOSCA1.6*, *GmOSCA2.1*, *GmOSCA2.6*, and *GmOSCA4.1*, which were extremely low expressed or possible pseudogenes, the remaining 16 *GmOSCA* genes were cloned. Currently, no such aequorin-based calcium imaging detection system is established in soybean as in *Arabidopsis*. In addition, the efficiency of soybean genetic transformation is lower than that of *Arabidopsis*, so it is hard to transform all *GmOSCA* genes into soybean. Therefore, we planned to investigate the functions of *GmOSCA* genes by overexpression of *GmOSCAs* in the *osca1* mutant. In addition, the CDS of *AtOSCA1.1* and the empty vector pfgc5941 were transformed into the *osca1* mutant, to generate transgenic *Arabidopsis AtOSCA1.1* and *pfgc*, as the positive and negative controls, respectively. By observing the calcium imaging phenotype under 600 mM sorbitol treatment, a lower OICI was observed in *osca1* than in the wildtype (WT) (Figure 4A). Meanwhile, we found that the decreased OICI in *osca1* was fully complemented in transgenic *Arabidopsis* lines carrying *GmOSCA1.1*, *GmOSCA1.2*, *GmOSCA1.3*, *GmOSCA1.4*, and *GmOSCA1.5* (Figure 4A), which are the closest homologs with *AtOSCA1.1* in cluster I. However, the transgenic *Arabidopsis* lines with other *GmOSCA* genes in clusters I, II, III, and IV did not rescue the reduced OICI in *osca1*. To exclude the influence of the difference in total aequorin, the total amount of aequorin in each sample was measured using a discharge solution, and used as an internal reference for quantification [15]. It further supported the above conclusion (Figure 4B). Taken together, *GmOSCA1.1*, *GmOSCA1.2*, *GmOSCA1.3*, *GmOSCA1.4*, and *GmOSCA1.5* might function in the osmotic stress sensing of soybean, similar to *AtOSCA1.1* in *Arabidopsis.*

### 2.5. Expression Profiles of GmOSCA Genes against Osmotic Stress

**Figure 5:**
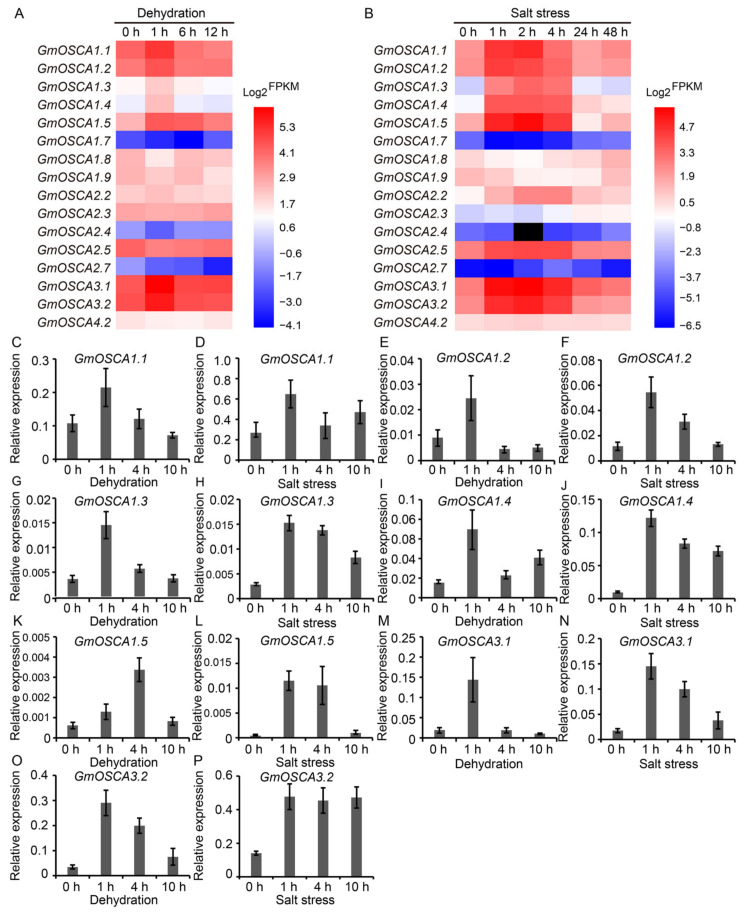
The expression patterns of *GmOSCA* genes in roots against dehydration and salt stress. (**A**) *GmOSCA*s expression profiles under dehydration using RNA-seq data. (**B**) *GmOSCA*s expression profiles under NaCl treatment using RNA-seq data. (**C**–**P**) *GmOSCA*s expression profiles in roots under PEG treatment and NaCl treatment using RT-qPCR data (mean ± SD, n = 3 or 4).

As potential osmosensors, the expressions of *GmOSCA* genes are likely to respond to osmotic stress. To support this hypothesis, the *GmOSCA*s expression profiles against dehydration and salinity (NaCl) treatments in soybean roots were explored, using the previously released Illumina RNA-seq raw data [25,26]. The expression profiles displayed that *GmOSCA1.1*, *GmOSCA1.2*, *GmOSCA1.3*, *GmOSCA1.4*, *GmOSCA1.5*, *GmOSCA3.1*, and *GmOSCA3.2* were significantly induced in the early stage (<6 h) of dehydration and salinity treatments and then gradually returned to normal with the extension of treatment time (Figure 5A,B). In contrast, *GmOSCA1.7*, *GmOSCA1.8*, and *GmOSCA2.4* were down-regulated only in the early stage of dehydration and salinity treatments, but these results may not be reliable because of the superficial expression level of *GmOSCA1.7* and *GmOSCA2.4* (Figure 5A,B). Interestingly, *GmOSCA2.7* was down-regulated under dehydration but up-regulated under salt stress (Figure 5A,B). However, this result was not very convincing because of the shallow expression level of *GmOSCA2.7*. In addition, *GmOSCA1.9* was down-regulated, but *GmOSCA2.2* and *GmOSCA2.5* were up-regulated by salt stress (Figure 5B). And there was little change in the expression of *GmOSCA4.2* under drought and salt stresses (Figure 5A,B). To further verify the response of *GmOSCA* genes to osmotic stress, RT-qPCR experiments were performed. The results showed that the gene expression of *GmOSCA1.1*, *GmOSCA1.2*, *GmOSCA1.3*, *GmOSCA1.4*, *GmOSCA1.5*, *GmOSCA3.1*, and *GmOSCA3.2* were indeed induced by drought and salt stresses in soybean roots (Figure 5C–P). Similarly, these genes were also up-regulated by drought and salt stress in soybean leaves (Supplementary Materials Figure S5). Taken together, our data demonstrated that most *GmOSCA* genes, especially *GmOSCA1.1*, *GmOSCA1.2*, *GmOSCA1.3*, *GmOSCA1.4*, *GmOSCA1.5*, *GmOSCA3.1,* and *GmOSCA3.2*, were osmotic-stress-responsive genes.

### 2.6. Overexpression of Some GmOSCA Members Complements the Drought-Hypersensitive Phenotype of osca1 Mutant

**Figure 6:**
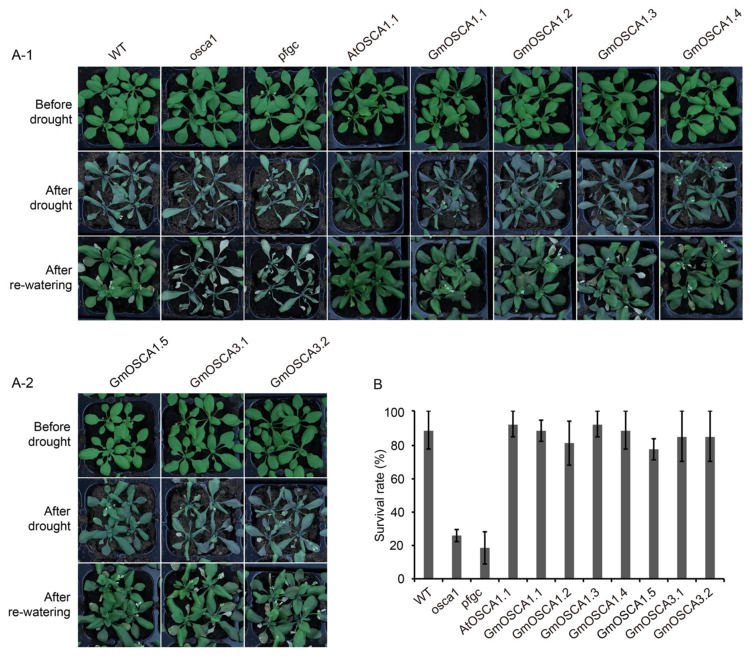
The drought-responsive phenotypes of *GmOSCA*s transgenic *Arabidopsis* plants. (**A**) Phenotypes of WT, *osca1,* and homozygous transgenic *Arabidopsis* lines. (**B**) The survival rates after re-watering were determined. Data were mean ± SE from three representative experiments.

Since *GmOSCA1.1*, *GmOSCA1.2*, *GmOSCA1.3*, *GmOSCA1.4*, and *GmOSCA1.5* were not only induced by osmotic stress but also could restore the low OICI phenotype of *osca1*, they were selected to further study the functions in response to osmotic stress at the whole-plant level. In addition, two other osmotic-stress-induced genes, *GmOSCA3.1* and *GmOSCA3.2*, also belonging to the early responsive to dehydration stress protein 4 (*ERD4*) family, were added for further functional studies too. We directly monitored the growth status of the WT, *osca1*, and transgenic *Arabidopsis* lines of *AtOSCA1.1*, *GmOSCA1.1*, *GmOSCA1.2*, *GmOSCA1.3*, *GmOSCA1.4*, *GmOSCA1.5, GmOSCA3.1*, *GmOSCA3.2*, and *pfgc* under drought stress in soil. During drought treatment, *osca1* and the empty *pfgc* transgenic plants exhibited earlier and more severe wilting than WT, whereas the *AtOSCA1.1*, *GmOSCA1.1*, *GmOSCA1.2*, *GmOSCA1.3*, *GmOSCA1.4*, *GmOSCA1.5*, *GmOSCA3.1*, and *GmOSCA3.2* transgenic lines could fully complement the drought-hypersensitive phenotype of *osca1* and showed the same or even stronger drought tolerance than WT (Figure 6A). After re-watering, the survival rates of *osca1* and the empty *pfgc* transgenic plants were much lower than that of WT, while the lower survival rate of *osca1* could be fully rescued by the overexpression of *GmOSCA1.1*, *GmOSCA1.2*, *GmOSCA1.3*, *GmOSCA1.4*, *GmOSCA1.5*, *GmOSCA3.1*, and *GmOSCA3.2*, just like *AtOSCA1.1* (Figure 6A,B). Despite the similar survival rates to WT, the restoring states of these transgenic *Arabidopsis* lines were different, among which some transgenic lines seemed to show better growth status than WT (Figure 6A).

## 3. Discussion

The signal transduction pathway of plant adaptation to osmotic stress, which seriously affects plant growth and crop yield, contains the processes of stress perception, signal transduction, and adaptive responses, such as stress-responsive gene expression and the production of metabolites. Compared with the well-studied processes of signal transduction and stress adaptation to osmotic stress, until we discover AtOSCA1.1, it is not clear who is the osmosensor responsible for osmotic sensing in plants. *AtOSCA1.1* belongs to the *OSCA* family, which contains four clades and 15 *OSCA* members in *Arabidopsis*. Among the 15 *AtOSCA* members, the two paralogs, AtOSCA1.1 and AtOSCA1.2, with the highest sequence identity are hyperosmolarity-gated calcium-permeable channels involved in osmotic stress signaling [15,16], suggesting that the *OSCA* family may be functionally conserved and redundant, especially for members with high similarity. Furthermore, with the resolution of the cryo-electron microscopy structures of AtOSCA1.1, AtOSCA1.2, AtOSCA2.2, and AtOSCA3.1 [27,28,29,30], we know that the OSCA family belongs to a new group of mechanosensitive ion channels, which have quite a similar topological structure as the mammalian TMEM16 proteins. The conformation changes of OSCA channels lead to the opening of the ion channel pore under mechanical stimuli, such as gravity, touch, and osmotic pressure, which triggers the local tension and deformation of the membrane. These similar cryo-electron microscopies of the OSCA family further support that they may have conserved protein functions. However, electrophysiological examinations of different subclasses of *OSCA* family members expressed in HEK-P1KO cells revealed quite a distinct mechanically activated ion conductance, implicating the divergent channel properties in OSCA family members with different clades [31]. In this study, we demonstrated that GmOSCA1.1, GmOSCA1.2, GmOSCA1.3, GmOSCA1.4, and GmOSCA1.5 derived from cluster I have functions similar to AtOSCA1.1, at least at the protein level, by the heterologous expression of *GmOSCA* members in *osca1* (Figure 4), supporting the concept that the *OSCA* family members with high identity are likely functionally conserved and redundant. In contrast, the fact that the remaining *GmOSCA* genes derived from clusters I, II, III, and IV did not rescue the reduced OICI of *osca1* suggests the different functional properties between them with *AtOSCA1.1* (Figure 4). Similarly, *AtOSCA3.1* is an early inducible gene in response to drought stress, and its knockout mutant displayed wildtype OICI under osmotic stress, which also supports a different biological working mechanism between *AtOSCA3.1* and *AtOSCA1.1* [15]. Recently, AtOSCA1.3 was demonstrated as a Ca^2+^-permeable channel required for stomatal immunity, and its activation depends on BIK1-mediated phosphorylation at its serine residue within a motif (Ser-X-X-Leu) [17]. Besides AtOSCA1.3, AtOSCA1.7 within OSCA cluster I also had a similar motif to AtOSCA1.3 at the same position and was activated by BIK1 activity. Furthermore, an *Atosca1*.3/*1.7* double mutant exhibited impaired stomatal closure and reduced flg22-induced Ca^2+^ increase upon treatment with flg22 [17]. Although it remains to be tested whether AtOSCA1.3 and AtOSCA1.7 are similarly mechanosensitive to other OSCAs, these results suggested that phosphorylation by BIK1 may represent an additional regulatory layer for this conserved family of Ca^2+^-permeable channels in response to different stresses. Therefore, we used sequence alignment to explore whether GmOSCA members of cluster I have a similar motif (Ser-X-X-Leu) and found that only GmOSCA1.5 had this motif (Supplementary Materials Figure S6), indicating that, in addition to sensing osmotic stress, it may also regulate soybean immunity.

Besides the model plant *Arabidopsis*, the *OSCA* gene family has been identified genome-wide and described in several plant species, but only a very few *OSCA* genes have been functionally validated, except those in rice, of which 11 *OsOSCA* members were identified and divided into four clades, like *Arabidopsis* and soybean. The functions of 11 *OsOSCA* members, except for *OsOSCA4.1*, have been reported in several papers [32,33,34]. Of the 10 OsOSCA members, only OsOSCA1.4 was exclusively located in the PM, while the other 9 OsOSCAs were mainly localized in the endoplasmic reticulum (ER). In addition, *OsOSCA1.4* mediated both OICI and salt-stress-induced cytosolic [Ca^2+^] increases (SICI_cyt_) in HEK293 cells and *osca1* mutant, suggesting that OsOSCA1.4 may function as an osmosensor in rice [34]. Surprisingly, although different from AtOSCA1.1 localization, overexpressing of some of the remaining *OsOSCA* genes (*OsOSCA1.1*, *OsOSCA1.2*, *OsOSCA1.3*, *OsOSCA2.1*, and *OsOSCA2.2*) could restore the reduced OICI and SICI_cyt_ in *osca1* [33]. Recently, the biological function of *OsOSCA1.1* in rice was reported and showed that *OsOSCA1.1* mediated OICI and SICI_cyt_ in rice roots under hyperosmolality and salt stress [32]. These results further proved that the *OSCA* family members are functionally conservative and specific.

Soybean, which provides more than one-half of global oilseed production and one-quarter of the world’s protein for humans and livestock, is susceptible to osmotic stress such as drought and salinity. Soybean-growing regions and production are seriously limited due to the two major threats of drought and salt stresses [35,36]. Therefore, cloning genes related to osmotic stress tolerance and studying their underlying molecular mechanisms are crucial for breeding new drought- or salt-tolerant soybean cultivars and improving soybean yield through molecular breeding techniques. In recent decades, especially after the release of the soybean genome, with the demand for agricultural production and the rapid development of molecular biology technology, some genes involved in the osmotic stress regulation in soybean have been cloned and reported. These genes are mainly transcription factors regulating the expression of downstream stress genes or stress-responsive genes themselves, which are downstream of the signal transduction pathway. In this study, we first systematically studied and experimentally demonstrated the functions of *GmOSCA* members, which may involve osmotic stress perception as osmosensors in soybean. A total of 20 *GmOSCA* members in four subgroups were identified in the soybean genome (Figure 1). The number of *OSCA* genes in soybean was significantly higher than that in diploid *Arabidopsis* (15) and rice (11), which is consistent with the fact that soybean is paleo-polyploid. In addition, our collinearity analysis showed that all of the *GmOSCA* genes, apart from *GmOSCA1.5* and *GmOSCA2.7*, are duplicated genes (Supplementary Materials Figure S2), which could provide a chance for gene evolution. On the one hand, soybean has more *OSCA* members, and, on the other hand, most of these genes are duplicated genes, indicating that they may have greater functional redundancy and diversity. The comparative analyses of homologs between soybean and *Arabidopsis* on gene structures and protein domains and structures suggest that the *GmOSCA* members in the same cluster may have similar functions, and *GmOSCA* genes in clusters III and IV may have generated divergent functions (Figure 1 and Supplementary Materials Figure S3). Our gene-expression data confirmed that *GmOSCA* members produced nonfunctionalization and subfunctionalization at gene expression. For instance, the spatial- and temporal-expression patterns of soybean *GmOSCA*s genes are very different, and some genes are even pseudogenes (Figure 2 and Supplementary Materials Figure S4). In addition, they also have different responses to drought and salt stress (Figure 5 and Supplementary Materials Figure S5). Finally, our complementary experiments in *osca1* showed that the *GmOSCA* family also produced functional differentiation at the protein level (Figure 4 and Figure 6).

Here, the presented data preliminarily proved that GmOSCA1.1, GmOSCA1.2, GmOSCA1.3, GmOSCA1.4, and GmOSCA1.5 are likely calcium-permeable osmosensors in soybean by heterologous expression of *GmOSCA* members in *osca1* (Figure 4), and the overexpression of *GmOSCA1.1*, *GmOSCA1.2*, *GmOSCA1.3*, *GmOSCA1.4*, *GmOSCA1.5*, *GmOSCA3.1*, and *GmOSCA3.2* conferred enhanced drought tolerance to transgenic *Arabidopsis* (Figure 6). Unexpectedly, it was found that these *GmOSCA* genes did not affect salt tolerance, due to the similar survival rates and above-ground growth status of wildtype, *osca1*, and *OSCA* transgenic plants under hydroponic NaCl (50 mM and 80 mM) treatment (data not shown). In fact, when the hydroponic NaCl concentration is below 50 mM or above 80 mM, the *Arabidopsis* seedlings will survive or die accordingly. One of the most likely explanations for this phenomenon is that these *OSCA* genes may act primarily on osmotic stress. Although high salinity can increase osmotic stress, it also causes ion toxicity to plants. The accumulation of Na^+^ and Cl^−^ in cells affects the absorption and transport of mineral elements and inhibits the activity of intracellular enzymes [37]. Recently, using a similar calcium-imaging-based genetic screen, we identified that *MOCA1*, encoding a glucuronosyltransferase for glycosyl inositol phosphorylceramide (GIPC) sphingolipids in PM, is involved in sensing salt-associated ionic stress [38]. The increase in [Ca^2+^]_i_, found to be induced by 200 mM NaCl, was lower in the *moca1* mutant, while the rise in [Ca^2+^]_i_ caused by 400 mM sorbitol was similar between *moca1* and the wildtype. These data distinguished the ionic effect from the osmotic effect of salt stress. Thus, when the concentration of NaCl is low, it is mainly ion stress that plays a role. With the increase in NaCl concentration until the *Arabidopsis* seedlings die due to ion toxicity, osmotic stress does not play a significant role; thereby, highly concentrated NaCl cannot reflect the function of *OSCA* genes to enhance osmotic stress tolerance. However, to elucidate the natural biological function of *GmOSCA* genes, we still need further study of these genes, by overexpressing or silencing them in soybean. Moreover, since *GmOSCA* members may be functionally redundant, it may be necessary to knock out multiple genes simultaneously to study their function. Fortunately, CRISPR/Cas9-mediated gene-editing technology provides a solution to this problem. In addition, nearly 1000 soybean resequencing materials have been released so far. The association analysis between *GmOSCAs* genotypes and drought or salinity tolerance phenotypes using these materials can not only further support whether they are involved in the regulation of drought stress but also screen out the excellent alleles of *GmOSCAs* for breeding. Despite these problems, our study is the first to explore the process of osmotic stress perception in soybean, so our data will lay the foundation for further study of this process and provide valuable genetic resources for the development of drought-tolerant soybean cultivars using genetic engineering and molecular breeding.

## 4. Materials and Methods

### 4.1. Identification and General Characterization of GmOSCA Family Members in Soybean

The 15 *Arabidopsis* OSCA protein sequences were used to blast in the soybean genome (Wm82.a2.v1) in Phytozome (https://phytozome.jgi.doe.gov/pz/portal.html, accessed on 15 August 2022). The Pfam tool (http://pfam.xfam.org/) was then used to identify the conserved domains of candidates, including the typical RSN1_7TM domain in OSCA proteins [39]. Finally, 20 GmOSCA candidates with high sequence identity and RSN1_7TM domain were screened out in assembled soybean chromosomes. Protparam (https://web.expasy.org/protparam/) was adopted to calculate the relative molecular masses and isoelectric points of GmOSCA proteins. A neighbor-joining phylogenetic tree of OSCA proteins was constructed by MEGA 6.0 with the Poisson model, 1000 bootstrap replications, and a complete deletion treatment for gaps/missing data [40]. The *OSCA* gene structures were drawn using GSDS 2.0 software (http://gsds.cbi.pku.edu.cn/) [41]. The three-dimensional structures of OSCA proteins were predicted using Phyre2 (http://www.sbg.bio.ic.ac.uk/phyre2/html/) [42]. The collinear blocks carrying *GmOSCA* genes in soybean were identified with the MCScanX toolkit [43], and the collinear relationships were drawn by Circos [44]. Transmembrane helices and subcellular localizations of GmOSCA proteins were predicted using MemPype (https://mu2py.biocomp.unibo.it/mempype/default/index) [45].

### 4.2. RNA-Seq Data Analysis

The spatiotemporal expression profiles of *GmOSCA* genes in 25 samples were obtained by mining the Illumina RNA-seq raw data released by Shen et al. [24]. The expression patterns of *GmOSCA* genes against dehydration and salinity treatments were detected by reanalysis of the Illumina RNA-seq raw data from Belamkar et al. [26] and Liu et al. [25], respectively. The short reads were mapped and aligned with the soybean reference genome Wm82.a2.v1 using HISAT [46]. The assembly and expression calculations of these transcripts were achieved by StringTie [47]. The mean fragments per kilobase of exon per million fragments mapped (FPKM) value was regarded as the gene expression value. The heat maps were visualized utilizing Heml, with the FPKM values as input data [48].

### 4.3. Plant Materials and Abiotic Stress Treatments

The soybean cultivar Williams 82 was used in this study. Soybean plants for gene cloning were grown outdoors during the sowing season. For abiotic stress treatments, soybean seeds with uniform size and harvest time were firstly germinated on moist sterile filter paper for four days in the dark at 25 °C. They then were transferred to half-strength Hoagland-modified nutrient solution (Coolaber Biotech, Beijing, China) in a growth chamber with a 12 h light/12 h dark photoperiod, 65%~75% relative humidity, and an ambient temperature of 28 °C. The nutrient solution was renewed every two days. When the unifoliolate leaves were fully opened, seedlings were transferred into half-strength Hoagland-modified nutrient solution supplemented with 10% (*w*/*w*) PEG and 0.9% (*w*/*w*; ~150 mM) NaCl, respectively. 10% PEG hydroponics can cause plant dehydration and thus simulate soil drought. The unifoliolate leaf and root tissues were harvested at 0, 1, 4, and 10 h after treatments, and two biological replicates and 10 plants per time point were maintained for each treatment.

The *Arabidopsis* WT (Col-0 constitutively expressing intracellular Ca^2+^ indicator aequorin, from M. Knight) [49], *osca1*, and all the transgenic plants in *osca1* background were grown in a greenhouse with a 16 h light/8 h dark photoperiod at 20~23 °C. The sterilized seeds were vernalized at 4 °C for three days, before being sown on a half-strength MS medium. For the drought treatment in soil, the WT, *osca1*, and homozygous transgenic seedlings were first grown on half-strength MS medium for about one week and then transferred into the weighed soil for two weeks. The remaining water in the pot’s base was poured out and then withheld until the *osca1* plants developed the wilting phenotypes. The survival rates were surveyed after re-watering with three biological replicates [50].

### 4.4. RNA Extraction, cDNA Synthesis, and RT-qPCR

Total RNA was extracted using an EasyPure Plant RNA Kit (TransGen Biotech, Beijing, China). cDNA synthesis was performed with a kit of TransScript One-Step gDNA Removal and cDNA Synthesis SuperMix (TransGen Biotech, Beijing, China) or HiScript III All-in-one RT SuperMix Perfect for qPCR (Vazyme Biotech, Nanjing, China). Real-time quantitative PCR (RT-qPCR) was performed using the BIO-RAD C1000 Touch Thermal Cycler PCR system and Applied Biosystems PowerUp SYBR Green Master Mix Kit (Thermo Fisher Scientific, Shanghai, China), in accordance with the instructions of the manufacturer, with a slight modification. The relative gene expression levels of *GmOSCA* genes were calculated from three or four replicates according to the 2^−ΔCT^ method, with a reference gene ACTIN. The primers used for RT-qPCR are listed in Supplementary Materials Table S2.

### 4.5. Subcellular Localization of GmOSCA proteins

The full-length CDS of *GmOSCAs* and *AtOSCA1.1* were amplified from the soybean and *Arabidopsis* cDNA, respectively. Then, CDS was cloned into the PucGFP vector without stop codons for fusion with a green fluorescent protein (GFP) tag at the C-terminus through enzymatic digestion and ligation. The empty PucGFP vector and these recombinant plasmids were transformed into the digested *Arabidopsis* protoplasts, as previously described [51]. The GFP fluorescence was detected by laser confocal microscopy. The amplified primers for PucGFP are listed in Supplementary Materials Table S3.

### 4.6. Construction of OSCA Transgenic Arabidopsis Lines

The full-length CDS of *GmOSCAs* and *AtOSCA1.1* were cloned into the pfgc5941 vector for genetic complementation assay through enzymatic digestion and ligation. The empty pfgc5941 vector and these recombinant plasmids were transformed into the *osca1* mutant, using the floral dip method with *Agrobacterium tumefaciens* GV3101 strain. Transgenic *Arabidopsis* lines were screened by basta spraying (50 mg/L) and PCR test, and the homozygous lines with single copy insertion were used for experimental analysis. The amplified primers for pfgc5941 are listed in Supplementary Materials Table S3.

### 4.7. Aequorin Bioluminescence-Based Ca^2+^ Imaging

[Ca^2+^]_i_ was detected using *Arabidopsis* plants expressing aequorin, as described previously [15]. Nine-day-old *Arabidopsis* seedlings were evenly sprayed with 6 mL of 10 μM coelenterazine (Prolume) per Petri dish (15 cm in diameter) and then placed in the dark at 22 °C for 12 h before imaging. The aequorin bioluminescence-based Ca^2+^ imaging was conducted using a Lumazone Pylon1300B system (Roper Scientific, Tuscon, AZ, USA) equipped with a cooled CCD camera in a light-tight box. A liquid nitrogen autofiller was connected to this system to maintain constant cooling. The camera was controlled by WinView/32 (Roper Scientific, Tuscon, USA) software. The plate was treated with 90 mL 600 mM sorbitol (Sigma-Aldrich, Shanghai, China), and the recording of luminescence (*L*) was started 10 s before treatment and collected for 5 min. The total aequorin luminescence (*L_max_*) was recorded for 3 min by discharging with 0.9 M CaCl_2_ in 10% (*v*/*v*) ethanol. The bioluminescence images were analyzed using ImageJ software. The [Ca^2+^]_i_ was measured according to the formula (pCa = 0.6747 × (−log L/Lmax) + 5.3177), and calculated from four replicates [15,38].

## 5. Conclusions

In this study, we identified 20 *GmOSCA* members in soybean and systematically compared their phylogenetic relationships, gene structures, protein domains and structures, spatiotemporal and osmotic-stressed expression profiles, and protein functions in transgenic *Arabidopsis*. The results showed that 20 *GmOSCA* members were divided into four clades and that the members in the same cluster may have more similar functions. In addition, *GmOSCA1.1*, *GmOSCA1.2*, *GmOSCA1.3*, *GmOSCA1.4*, and *GmOSCA1.5* from cluster I might function in the osmotic stress sensing of soybean. Furthermore, *GmOSCA1.1*, *GmOSCA1.2*, *GmOSCA1.3*, *GmOSCA1.4*, *GmOSCA1.5*, *GmOSCA3.1*, and *GmOSCA3.2* might confer enhanced drought tolerance in soybean. These results greatly promote the research progress of the *GmOSCA* family, enrich the molecular mechanism of how soybean responds to osmotic stress, and lay a foundation for improving soybean drought tolerance.

## Data Availability

Not applicable.

## Supplementary Materials

The following supporting information can be downloaded at: https://www.mdpi.com/article/10.3390/ijms231810570/s1.
