# Supplementary material for: Systematic Characterization of the OSCA Family Members in Soybean and Validation of Their Functions in Osmotic Stress
Source: Int J Mol Sci. 2022 Sep 12;23(18):10570. doi: 10.3390/ijms231810570 (PMC9500692; doi:10.3390/ijms231810570)
Supplement: Supplementary file 1 [file ijms-23-10570-s001.zip › ijms-1896396-supplementary/supplementary files/Supplementary Figure S1-S6.pdf]

Percent identity

Divergence

	1	2	3	4	5	6	7	8	9	10	11	12	13	14	15	16	17	18	19	20		
1		98.5	85.8	85.7	75.0	65.8	65.8	59.0	57.9	30.1	30.4	35.1	34.0	30.5	29.0	30.6	30.5	29.8	17.8	18.8	1	GmOSCA1.1
2	1.6		86.0	85.9	75.4	66.0	66.0	58.6	57.5	29.9	29.9	34.7	33.7	30.1	28.9	30.7	30.3	29.6	17.9	19.0	2	GmOSCA1.2
3	15.8	15.5		96.5	72.8	66.3	66.3	58.5	57.7	29.7	30.9	34.1	33.3	29.3	27.8	30.5	31.0	30.5	17.6	18.3	3	GmOSCA1.3
4	16.0	15.6	3.6		73.1	66.8	66.3	57.7	56.8	29.7	30.7	33.7	32.5	29.2	27.8	30.2	31.3	30.6	17.0	18.1	4	GmOSCA1.4
5	30.4	29.9	33.9	33.3		66.4	66.5	61.7	61.5	29.0	28.7	32.6	31.8	29.8	29.4	30.1	31.5	30.9	19.5	20.2	5	GmOSCA1.5
6	45.5	45.2	44.6	43.6	44.5		94.4	58.8	58.3	28.1	28.3	32.6	31.9	28.1	27.3	28.4	28.8	28.5	17.3	18.3	6	GmOSCA1.6
7	45.5	45.2	44.6	44.6	44.2	5.8		59.2	58.7	28.3	28.7	32.7	32.1	28.8	27.7	29.3	29.6	29.3	17.9	19.0	7	GmOSCA1.7
8	58.5	59.4	59.7	61.4	53.1	59.0	58.2		94.6	29.2	29.8	33.4	32.8	30.6	30.1	31.5	31.2	30.8	18.8	18.1	8	GmOSCA1.8
9	61.0	61.9	61.4	63.4	53.6	60.2	59.3	5.6		28.9	29.4	32.4	32.5	30.1	29.7	30.8	31.8	31.4	18.6	18.1	9	GmOSCA1.9
10	159.5	160.6	161.9	161.9	166.5	172.7	171.7	165.2	167.1		74.1	42.0	42.5	43.6	41.4	34.2	26.0	26.0	14.5	14.1	10	GmOSCA2.1
11	157.7	160.8	154.2	155.8	168.9	171.6	168.6	161.2	163.9	31.8		43.5	43.3	44.7	41.4	35.6	26.8	26.6	14.6	13.3	11	GmOSCA2.2
12	132.2	133.8	137.0	139.2	144.9	144.9	144.1	140.4	145.6	104.0	99.0		77.7	62.1	58.6	36.7	29.1	29.2	16.3	15.0	12	GmOSCA2.3
13	137.3	138.9	140.9	145.4	149.0	148.5	147.7	143.6	145.1	102.4	99.7	26.5		60.9	58.1	36.3	26.8	26.8	16.8	14.8	13	GmOSCA2.4
14	157.1	159.1	164.4	165.3	161.0	172.9	167.9	156.5	159.1	98.8	95.3	52.3	54.8		86.9	36.9	27.8	27.7	16.7	16.4	14	GmOSCA2.5
15	166.5	166.9	175.0	175.0	163.8	179.2	176.1	159.4	162.0	106.2	106.5	59.5	60.4	14.5		37.6	27.1	27.1	15.9	15.9	15	GmOSCA2.6
16	156.2	155.6	157.0	158.8	159.3	170.5	164.6	150.9	155.0	136.5	130.0	124.9	126.6	124.1	121.0		27.1	27.0	16.1	14.7	16	GmOSCA2.7
17	157.0	158.1	153.6	151.9	150.9	167.9	162.3	152.4	149.2	189.3	182.4	165.7	182.9	175.0	180.3	180.3		96.8	16.8	17.1	17	GmOSCA3.1
18	161.5	162.7	157.0	156.2	154.2	170.0	164.2	155.0	151.7	189.0	184.4	165.5	182.6	176.1	180.3	181.2	3.3		16.5	16.6	18	GmOSCA3.2
19	277.0	276.0	279.0	289.0	253.0	284.0	276.0	262.0	264.0	342.0	337.0	303.0	294.0	296.0	309.0	307.0	294.0	298.0		91.4	19	GmOSCA4.1
20	261.0	258.0	268.0	271.0	244.0	268.0	258.0	271.0	271.0	352.0	374.0	330.0	332.0	301.0	309.0	337.0	289.0	296.0	9.2		20	GmOSCA4.2
	1	2	3	4	5	6	7	8	9	10	11	12	13	14	15	16	17	18	19	20		

Figure S1. The sequence identity analysis of GmOSCA proteins. The values of protein identity were calculated using MegAlign software.

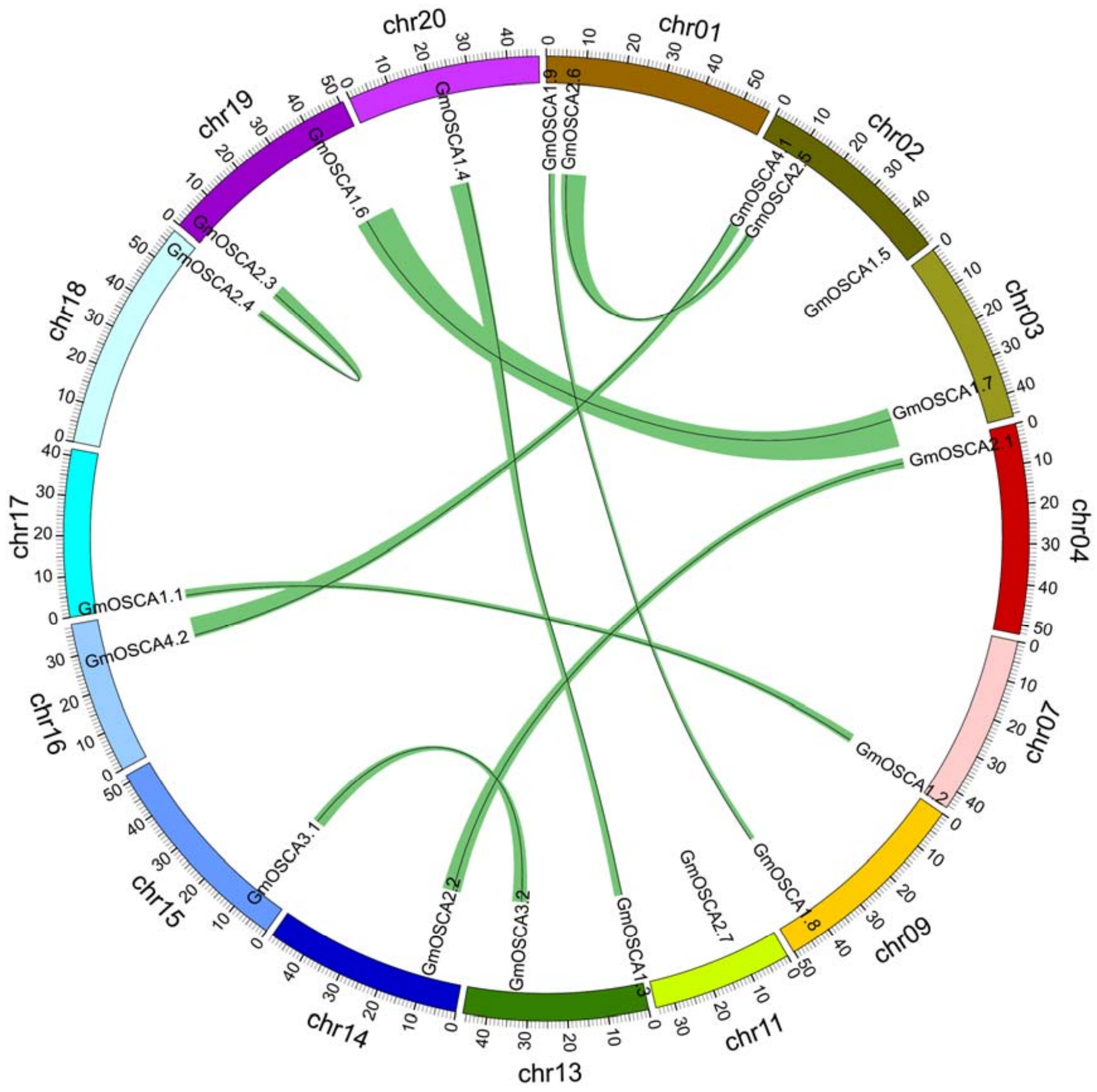

Figure S2. The collinear relationships of homologous blocks containing *GmOSCA* genes. The green-colored rainbows indicated the syntenic blocks. The black lines within these rainbows represented the location of *GmOSCA* genes.

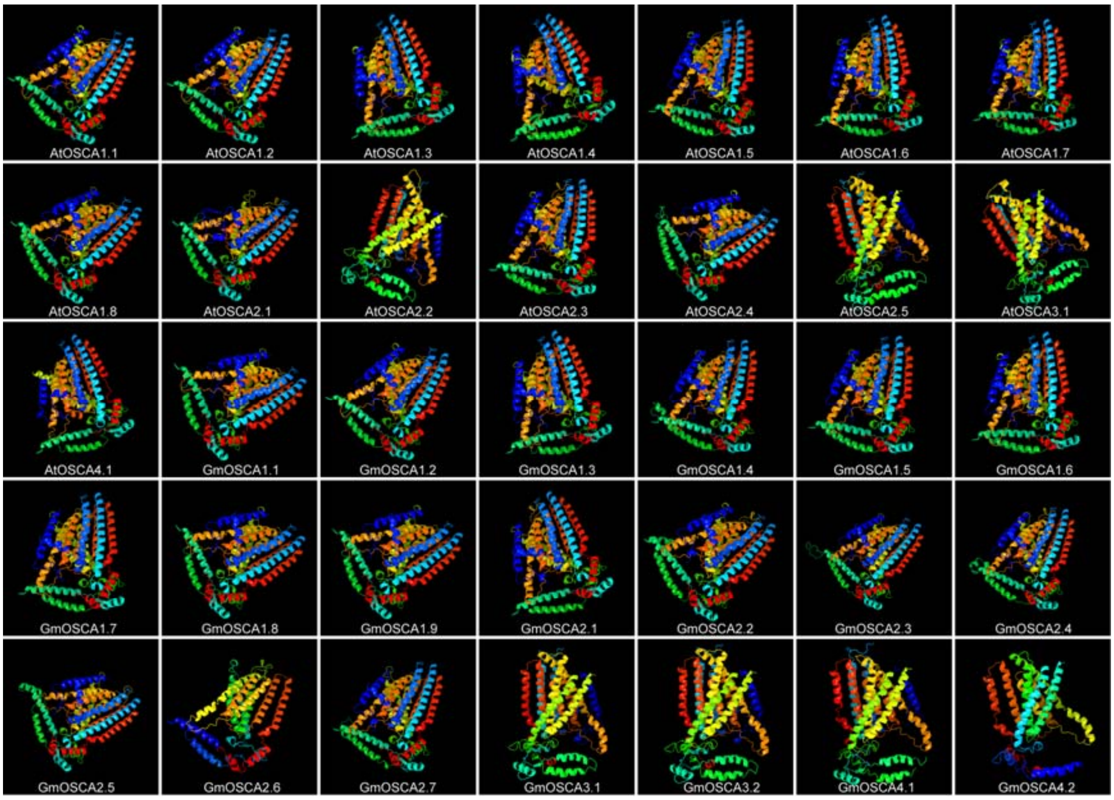

Figure S3. The three-dimensional structures of OSCA proteins from *Arabidopsis* and soybean.

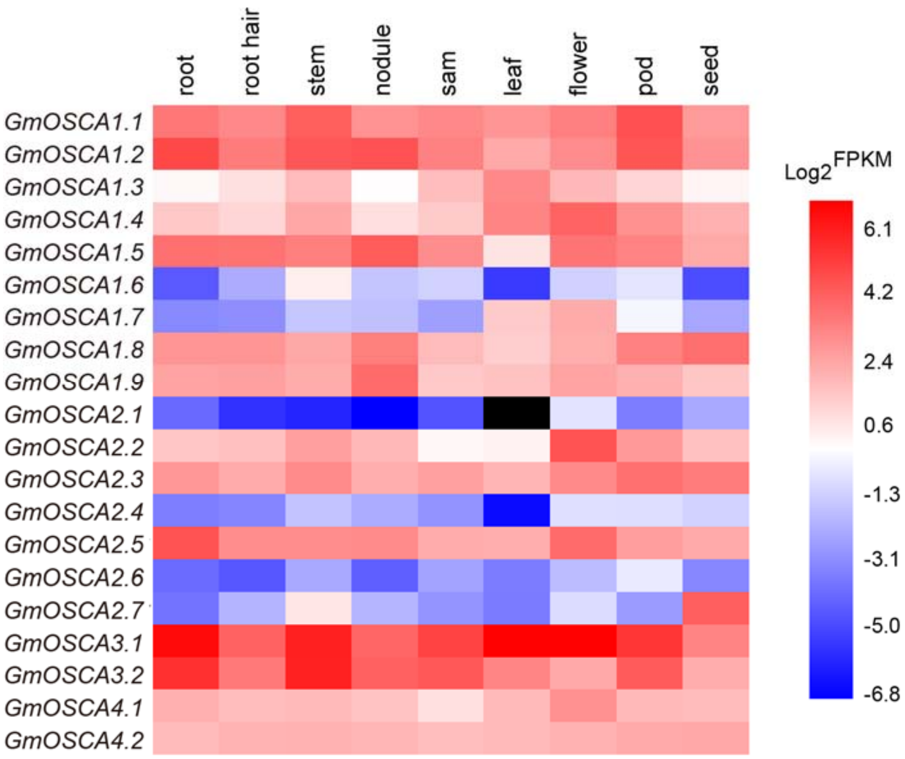

Figure S4. The tissue expression profiles of *GmOSCA* genes in soybean. Gradient color blocks represented log2 transformed FPKM values. The black blocks indicated that no FPKM value was available.

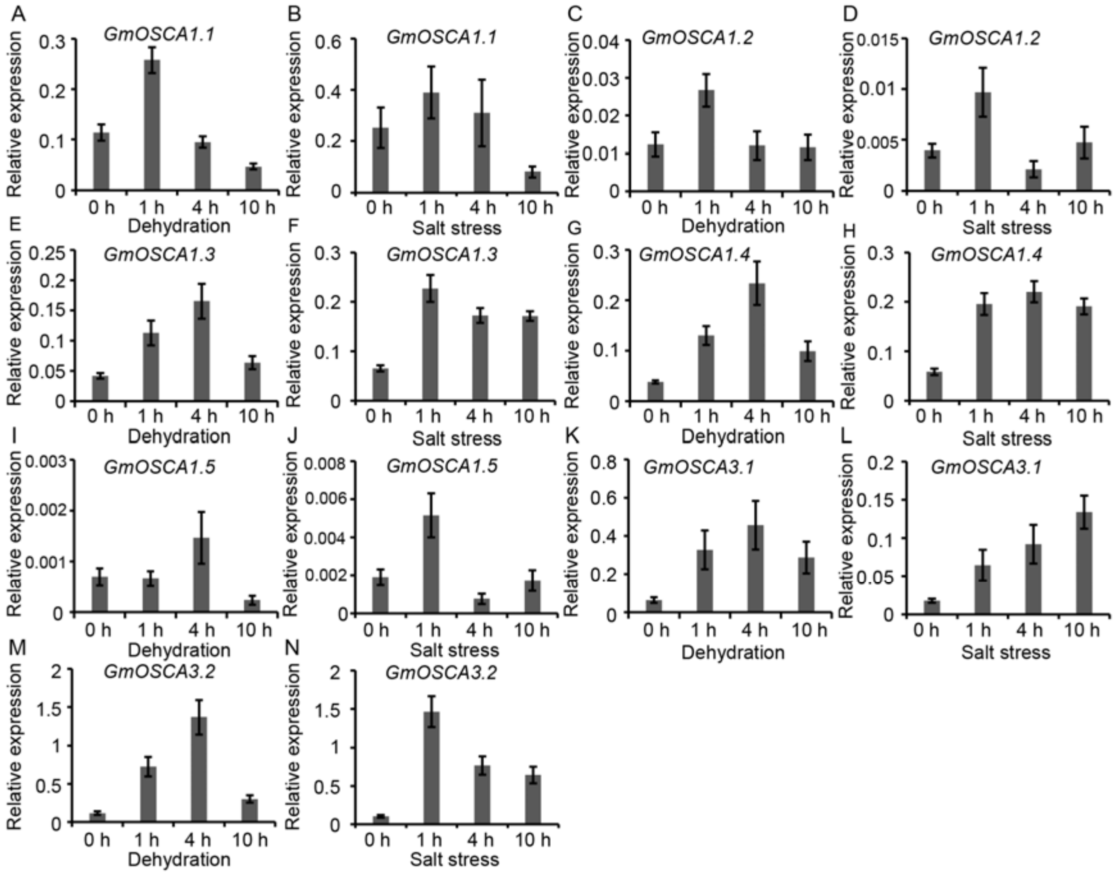

Figure S5. The expression patterns of *GmOSCA* genes in leaves against dehydration and salt stress.

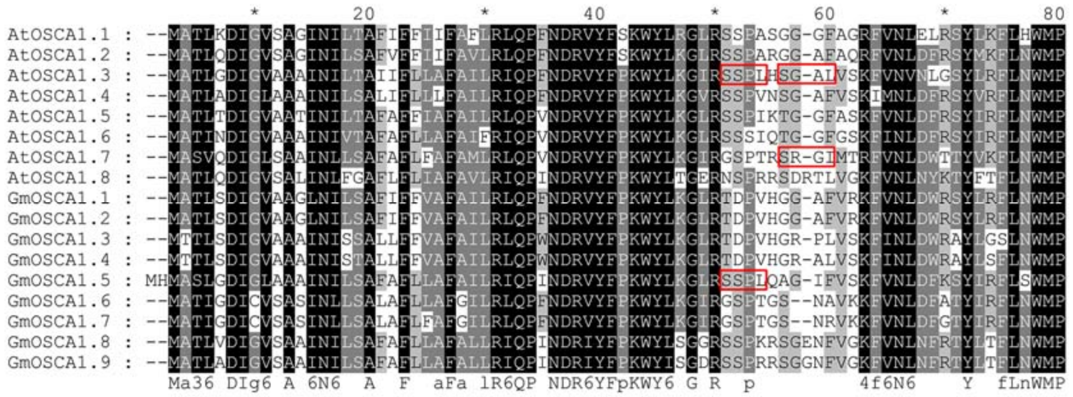

Figure S6. Protein sequence alignment of OSCA members in cluster I from *Arabidopsis* and soybean. Possible BIK1 phosphorylation motifs (SxxL/I) were highlighted in the red box.
